# A novel biopsy scheme for prostate cancer: targeted and regional systematic biopsy

**DOI:** 10.1186/s12894-024-01461-4

**Published:** 2024-04-13

**Authors:** Yang He, Yu Fan, Haitian Song, Qi Shen, Mingjian Ruan, Yuke Chen, Derun Li, Xueying Li, Yi Liu, Kai Zhang, Qian Zhang

**Affiliations:** 1 Department of Urology, The Institute of Urology, Peking University First Hospital, Peking University, The National Urological Cancer Center of China, No. 8 Xishiku St., Xicheng District, Beijing, 100034 China; 2 Institution of Urology, PekingUniversity, Beijing, 100034 China; 3 Beijing Key Laboratory of Urogenital Diseases (Male) Molecular Diagnosis and Treatment Center, Beijing, 100034 China; 4 National Urological Cancer Center, Beijing, 100034 China; 5 Department of Statistics, Peking University First Hospital, Beijing, China

**Keywords:** Prostate cancer, Biopsy scheme, Radical prostatectomy, Regional systematic biopsy, Targeted biopsy

## Abstract

**Purpose:**

To explore a novel biopsy scheme for prostate cancer (PCa), and test the detection rate and pathological agreement of standard systematic (SB) + targeted (TB) biopsy and novel biopsy scheme.

**Methods:**

Positive needles were collected from 194 patients who underwent SB + TB (STB) followed by radical prostatectomy (RP). Our novel biopsy scheme, targeted and regional systematic biopsy (TrSB) was defined as TB + regional SB (4 SB-needles closest to the TB-needles). The McNemar test was utilized to compare the detection rate performance for clinical significant PCa (csPCa) and clinical insignificant PCa (ciPCa). Moreover, the accuracy, positive predictive value (PPV) and negative predictive value (NPV) were investigated. The agreement between the different biopsy schemes grade group (GG) and RP GG were assessed. The concordance between the biopsy and the RP GG was evaluated using weighted κ coefficient analyses.

**Results:**

In this study, the overall detection rate for csPCa was 83.5% (162 of 194) when SB and TB were combined. TrSB showed better NPV than TB (97.0% vs. 74.4%). Comparing to STB, the TB-detection rate of csPCa had a significant difference (*p* < 0.01), while TrSB showed no significant difference (*p* > 0.999). For ciPCa, the overall detection rate was 16.5% (32 of 194). TrSB showed better PPV (96.6% vs. 83.3%) and NPV (97.6% vs. 92.9%) than TB. Comparing to STB, the detection rate of both schemes showed no significant difference (*p* = 0.077 and *p* = 0.375). All three schemes GG showed poor agreement with RP GG (TB: 43.3%, TrSB: 46.4%, STB: 45.9%). Using weighted κ, all three schemes showed no difference (TB: 0.48, TrSB: 0.51, STB: 0.51). In our subgroup analysis (PI-RADS = 4/5, *n* = 154), all three schemes almost showed no difference (Weighted κ: TB-0.50, TrSB-0.51, STB-0.50).

**Conclusion:**

Our novel biopsy scheme TrSB (TB + 4 closest SB needles) may reduce 8 cores of biopsy compared with STB (standard SB + TB), which also showed better csPCa detection rate than TB only, but the same as STB. The pathological agreement between three different biopsy schemes (TB/TrSB/STB) GG and RP GG showed no difference.

**Supplementary Information:**

The online version contains supplementary material available at 10.1186/s12894-024-01461-4.

## Background

Globally, prostate cancer (PCa) affects millions of men, with varying biological characteristics, thus increasing the complexity of its diagnosis and treatment. Therefore, it is crucial to harness histopathological grading as a tool to predict treatment response and outcome. The US Preventive Services Task Force [1] and European Association of Urology (EAU) [2] have recommended transrectal ultrasonography (TRUS)-guided systematic biopsy as the standard procedure to report histopathological grading for PCa. Ideally, a TRUS-guided biopsy involves the use of approximately 10–12 biopsy needles obtained systematically from the prostate. However, a TRUS-guided systematic biopsy (SB) may miss several PCa foci. With the development of multiparametric magnetic resonance imaging (mpMRI), the use of MRI-ultrasound (US) fusion for targeted biopsy (TB) has risen significantly over the last decade. According to previous investigations [3–5], MRI-TB could decrease the misidentification of the stages of PCa in males with visible lesions on MRI. Michael et al. [6] found that combined biopsy (SB + TB) diagnosed more patients with PCa than TB or SB alone, providing an effective method to characterize the extent of the disease.

As per the EAU guidelines, standard biopsy (STB, SB + TB) remains an effective approach for diagnosing PCa and has gained worldwide recognition. In STB, both the ipsilateral and contralateral lobes are biopsied in a predominantly random, systematic fashion, which is unique among solid-organ cancer diagnostic processes [7, 8]. However, increased sampling of the index lesion (perilesional/regional), while diagnostically beneficial for most men, can also result in unnecessary biopsy cores, potential harms, and patient burdens, as indolent cancers in men can be detected with false-positive MRI scans [9–11]. This raises concerns about the concept of STB and raises the question of whether SB can be reduced from 10 to 12 to fewer needles without compromising diagnostic accuracy. Recently, research has explored MRI-TB combined with only regional SB as an optimized approach to reduce biopsy cores [12–16]. Additionally, terms like focal saturation biopsy, perilesional biopsy, regional TB, and targeted sector biopsy have appeared in the literature [14, 15, 17–20]. All these studies aimed to reduce the number of unnecessary SB cores while maintaining detection rates for clinically significant PCa (csPCa). A systematic review, for example, found no significant difference between TB + regional SB and standard TB + SB [21].

Several key questions remained unanswered regarding the novel biopsy scheme: defining “regional”, optimizing the number of systematic needles and cores, and evaluating its pathological agreement with the established radical prostatectomy Grade Group (GG) system. To address these uncertainties, our study redefined the *Targeted and Regional Systematic Biopsy* (TrSB) scheme as TB combined with the 4 systematic biopsy needles closest to the targeted lesions. We then assessed both the detection rate and pathological GG concordance of this refined approach compared to the standard radical prostatectomy GG.

## Methods

### Patient population

A total of 505 patients underwent RP at our institution between December 2021 and March 2023. Patients who also underwent prior corresponding biopsies (SB + TB simultaneously) were re-reviewed retrospectively. The exclusion criteria were as follows: (1) Prostrate Imaging-Reporting and Data System, v2.1 (PI-RADS) score $\le$2 (*n* = 49); (2) no MRI data from our institution (*n* = 198); (3) prior hormonal therapy or radiotherapy (*n* = 25); and (4) prostate-specific antigen (PSA) >20 ng/mL (*n* = 39). Of 505 patients, 311 were excluded, and 194 were included in the final analysis. This retrospective observational study was approved by the Institutional Review Board of the Peking University First Hospital (protocol code 2016 − 1252).

### Clinicopathological characteristics

Patient clinicopathological characteristics including age, pre-operative PSA level, free PSA (fPSA)/total PSA (tPSA), prostate volume (PV) measured by TRUS, PSA density, PI-RADS score, Gleason score (GS), GG following prostate biopsy, and pathological characteristics of specimens following RP were collected.

### mpMRI and biopsy procedure

MRI was performed on a 3.0T whole-body system (GE Healthcare, USA) with no endorectal coil. The imaging protocol included axial T1-weighted images of the pelvis and biplanar T2-weighted fast spin-echo images centered on the prostate. Axial diffusion-weighted imaging was performed using b-values of 0, 800, and 1400 s/mm^2^. Dynamic contrast-enhanced images were obtained following intravenous administration of gadolinium chelate. Targets identified on MRI were scored by uroradiologists using the PI-RADS v2.1 scale from 1 (no findings suspicious for cancer) to 5 (very high probability). One of three experienced urologists with more than 5 years of expertise in prostate biopsy performed transrectal approaches to take TB + SB from patients in our group while under local anesthesia (Yi Liu and Derun Li). 30 min prior to the biopsy process, an oral antibiotic was given, none of biopsy complications happened in our biopsy procedure, as Clavien-Dindo systems classified. All patients underwent 10–12-core TRUS-guided SB and 2–4-core MRI-TB at our institution. All prostate cases were reported by two genitourinary pathologists following standardized reporting protocols.

### Scoring methods and definition

The 2005 International Society of Urological Pathology (ISUP) modified Gleason grading system, initially known as the global Gleason score (GS), assigns a grade based on the sum of the predominant and highest Gleason pattern scores identified within a prostate biopsy sample. The new 2016 ISUP GS grading system classifies PCa into the following five grades: grade group 1, GS 6; grade group 2, GS 3 + 4 = 7; grade group 3, GS 4 + 3 = 7; grade group 4, GS 4 + 4 = 8; and grade group 5, GS 9 and 10. Grades were separately reported for each positive needle. In general, the pathologists assessed all positive needles from the biopsies without giving a different weight to the two biopsies (SB and TB). A dedicated uropathologist (Qi Shen, with > 10 years of experience) performed the histopathological assessment. csPCa was defined as GS $\ge$3 + 4, equivalent to GG 2–5. Clinical insignificant PCa (ciPCa) was defined as GS = 3 + 3 (GG 1). In this study, the term “TrSB” refers to a targeted biopsy scheme incorporating four SB needles positioned in closest proximity to the TB needles.

### Scores for RP

The pathological handling of the RP specimen was performed according to the ISUP recommendation and CAP standardized protocol (College of American Pathologist protocol, v3.3.0.0., 2017), which included standardized sectioning and submitting the entire prostate gland. The GG in the RP was assigned for each case.

### Statistical analysis

Variables were reported as mean and standard deviation. Count and proportion (%) were used to report categorical variables. The paired chi-square test (McNemar test) was utilized to compare the detection performance for PCa, csPCa, and ciPCa. Moreover, the accuracy, positive predictive value (PPV), and negative predictive value (NPV) were investigated. The agreement between the different biopsy schemes GG and RP GG was assessed. The concordance between the biopsy and the RP GG was evaluated using weighted κ coefficient analyses (κ agreement 0: agreement is weaker than expected by chance; 0.01–0.20: slight agreement; 0.21–0.40: fair agreement; 0.41–0.60: moderate agreement; 0.61–0.80: substantial agreement; and 0.81–0.99: almost perfect agreement).

## Results

### Demographic and clinical characteristics

Between December 2021 and March 2023, our institution enrolled 194 patients who underwent SB, MRI-US cTB, and subsequent RP. Baseline clinical and MRI characteristics are detailed in Table 1. The mean patient age was 66.0 years (range: 48–78 years). Pre-operative PSA levels averaged 10.3 ng/mL (range: 2.5–20.0 ng/mL). The mean free PSA/total PSA ratio was 0.18 (range: 0.04–0.60 ng/mL). Prostate volume (PV) averaged 44.5 mL^3^ (range: 17.0-159.5 mL^3^), and the mean PSA density was 0.27 (range: 0.04–1.03). The mean MRI PI-RADS score was 4.2 (range: 3–5, PI-RADS 3: 40, PI-RADS 4: 83, PI-RADS 5: 71). An average of 2.3 targeted biopsy needles were utilized (range: 2–4), with a mean of 1.7 needles (range: 0–4) detecting tumor (TBx-positive), resulting in a 77.8% positive ratio. On average, 14.3 systematic biopsy needles were used (range: 14–16), with a mean of 4.6 needles (range: 1–14) detecting tumor (STBx-positive), for a 28.1% positive ratio. Our novel TrSB biopsy scheme employed a mean of 6.3 needles (range: 6–8), with a mean of 3.6 needles (range: 0–6) detecting tumors, yielding a 58.0% positive ratio. Importantly, TrSB required 8 fewer needles than the standard STB approach. Finally, the RP specimens revealed the following GG distributions: GG1 (*n* = 10), GG2 (*n* = 82), GG3 (*n* = 49), GG4 (*n* = 24), and GG5 (*n* = 29).

**Table 1:** Clinical and imaging characteristics at biopsy (194 Patients)

	Mean	SD	Range
**Age (y)**	66.0	6.0	48–78
**PSA (ng/mL)**	10.3	4.4	2.5–20.0
**fPSA/tPSA**	0.18	0.1	0.04–0.6
**Prostate volume (mL**^**3**^) **PSA density**	44.5 0.27	19.4 0.17	17.0-159.5 0.04–1.03
**PI-RADS score**	4.2	0.7	3–5
**No. TB needles** No. positive needles Positive-ratio	2.3 1.7 77.8%	0.6 0.8 0.3	2–4 0–4 0-100%
**No. STB needles** No. positive needles Positive-ratio	14.3 4.6 28.1%	0.6 0.3 0.1	14–16 1–14 23.5-28.6%
**No. TrSB needles** No. positive needles Positive-ratio	6.3 3.6 58.0%	0.6 1.4 0.2	6–8 0–6 0-100%

PSA indicates prostate-specific antigen; fPSA indicates free PSA; tPSA indicates total PSA; PI-RADS indicates Prostrate Imaging- Reporting and Data System; TB indicates targeted biopsy; STB indicates standard biopsy; TrSB indicates targeted and regional systematic biopsy

### csPCa and ciPCa detection rate

The overall detection rate for csPCa was 83.5% (162/194) when TB and SB were combined. As for patients with csPCa, GG2, GG3, GG4, and GG5 accounted for 42.0% (68/162), 30.2% (49/162), 16.7% (27/162), and 11.1% (18/162), respectively. The detection performance of different biopsy approaches was compared using systematic biopsy as the gold standard. As shown in Table 2, targeted biopsy missed nearly 11 cases of csPCa, while the novel TrSB scheme missed only 1. Both approaches exhibited high accuracy (94.3% and 99.5%, respectively) and PPV (100% for both). However, TrSB displayed a significantly superior NPV compared to TB (97.0% vs. 74.4%). Notably, the TB-detection rate of csPCa differed significantly from STB (*p* < 0.01), whereas TrSB showed no significant difference (*p* > 0.999). These findings suggest that TrSB offers superior detection performance for csPCa compared to both TB and STB.

**Table 2:** Comparison to standard biopsy for detection performance

	csPCa, *n* (%)	Accuracy (%)	PPV (%)	NPV (%)	P*
***STB***	162 (83.5)				
**TB**	151 (77.8)	94.3	100	74.4	< 0.01
**TrSB**	161 (83.0)	99.5	100	97.0	**> 0.999**
	**ciPCa, n (%)**				
***STB***	32 (16.5)				
**TB**	20 (10.3)	91.8	83.3	92.9	**0.077**
**TrSB**	28 (14.4)	97.4	96.6	97.6	**0.375**

csPCa indicates clinically significant prostate cancer, ciPCa indicates clinically insignificant prostate cancer, PPV indicates positive predictive value, NPV indicates negative predictive value, TB indicates targeted biopsy, TrSB indicates targeted and regional systematic biopsy

*Comparison to standard biopsy (SB + TB) for detection performance, McNemar test

Regarding ciPCa, the overall detection rate across both biopsy schemes was 16.5% (32 out of 194 patients). TB failed to detect 12 cases of ciPCa, while the novel TrSB scheme missed only 4 cases. Both approaches exhibited high accuracy (91.8% and 97.4%, respectively). Notably, TrSB demonstrated a superior PPV of 96.6% compared to TB’s 83.3% and a similarly superior NPV of 97.6% versus 92.9%. While neither TB nor TrSB showed significant differences in ciPCa detection rate compared to the standard systematic biopsy (*p* = 0.077 and *p* = 0.375, respectively), these findings suggest that TrSB may offer comparable detection performance for ciPCa with improved diagnostic specificity.

### Pathological score agreement between biopsy GG and RP GG

We tested the agreement between the GG assigned by three different biopsy schemes and the GG assigned by RP. As shown in Table 3, all three schemes showed moderate agreement (TB 43.3%, TrSB 46.4%, and STB 45.9%). Using weighted κ, there were no significant differences between the schemes (TB 0.48, TrSB 0.51, and STB 0.51). Compared to the RP GG group, the TB GG group exhibited downgrading in 14.9% and upgrading in 41.8% of cases, the TrSB group exhibited downgrading in 16.0% and upgrading in 37.6% of cases, and the STB group demonstrated downgrading in 16.5% and upgrading in 37.6% of cases.

**Table 3:** Weighted agreement and score changes between RP GG and three biopsy schemes GG

	**RP GG**						**Agreement**	**Weighted κ**	**Downgrade**	**No**	**Upgrade**
	**1**	**2**	**3**	**4**	**5**	**Total**	**(%)**	**(95% CI)**		**Change**	
**TB GG**							43.3	0.48 (0.41–0.55)	14.9%	43.3%	41.8%
0	7	11	1	0	0	19			0	0	19
1	2	18	4	0	0	24			0	2	22
2	1	35	10	4	2	52			1	35	16
3	0	14	28	7	3	52			14	28	10
4	0	4	4	9	14	31			8	9	14
5	0	0	2	4	10	16			6	10	0
**TrSB GG**							46.4	0.51 (0.43–0.58)	16.0%	46.4%	37.6%
0	2	1	0	0	0	3			0	0	3
1	5	22	3	0	0	30			0	5	25
2	3	41	15	4	1	64			3	41	20
3	0	14	25	7	5	51			14	25	12
4	0	4	3	9	13	29			7	9	13
5	0	0	3	4	10	17			7	10	0
**STB GG**							45.9	0.51 (0.44–0.58)	16.5%	45.9%	37.6%
0	0	0	0	0	0	0			0	0	0
1	7	23	2	0	0	32			0	7	25
2	3	43	17	4	1	68			3	43	22
3	0	14	22	8	5	49			14	22	13
4	0	2	5	7	13	27			7	7	13
5	0	0	3	5	10	18			8	10	0

RP indicates radical prostatectomy; GG indicates Grade Group; TB indicates targeted biopsy; TrSB indicates targeted and regional systematic biopsy; STB indicates standard biopsy(systematic and targeted); CI indicates confidence interval

In addition, we analyzed agreement within the subgroup of patients with PI-RADS scores of 4 or 5 (*n* = 154), as shown in the Supplementary Table. No significant difference was observed among the three schemes (weighted κ values of 0.50 for TB, 0.51 for TrSB, and 0.50 for STB). When compared to the RP GG group, the TB GG group exhibited downgrading in 16.2% and upgrading in 36.4% of cases, the TrSB GG group demonstrated downgrading in 16.9% and upgrading in 35.0% of cases, and the STB GG group had downgrading in 17.5% and upgrading in 36.4% of cases.

## Discussion

Biopsy remains the standard procedure for the diagnosis of PCa. A few decades ago, TRUS-guided biopsy using 10–12 needle schemes was considered the standard approach for men with suspected PCa [22]. While TRUS offers the advantage of being fairly sensitive and being able to perform during a clinic visit, it has limitations, including the under-detection of csPCas and over-detection of ciPCas [23]. An innovative approach to this diagnosis has been offered by mpMRI [24]. TB using MRI-US cognitive has been increasingly used recently in the diagnosis and characterization of PCa. There is an increasing consensus suggesting that MRI-TB could detect more intermediate/high-risk cancers using fewer biopsy needles while reducing the over-detection of low-risk cancer [25–30]. Furthermore, TB may offer a lower risk of Gleason score upgrades at radical prostatectomy, suggesting improved disease characterization [31–33]. However, many studies found that using SB or TB alone may have certain limitations [34–36]. While standard 10–12 needle template-guided prostate mapping biopsies (SB) target specific zones, they may miss some PCa foci. Similarly, MRI-TB focuses on MRI-positive areas, potentially overlooking lesions outside those zones. Combining SB and TB in a “TrSB” approach offers greater adaptability by addressing the limitations of each technique individually [37].

In recent years, MRI-directed biopsy approaches using TB and RSB (fewer cores than 10–12 needles) have been explored as an alternative approach to minimize biopsy cores, targeting errors, and grade migration. Marinus et al. summarized that the novel approach significantly improved lesion detection, tumor characterization, and tumor volume estimation and reduced the total number of biopsy cores and false-positive MRI results, potentially reducing procedure time and pathologist workload [21]. Furthermore, it is possible to reduce overdiagnosis rates by limiting SB to the area of the MRI-positive lesion. In addition, studies have suggested a 5–19% reduction in GG 1 PCa detection because fewer SB cores reduce the chance of finding indolent PCa in men with false-positive MRI scans [38, 39]. The reduction in biopsy cores did not significantly affect histopathological concordance. Nonetheless, a substantial number of men remain at risk of grade migration; biopsy cores underestimate or overestimate the true GS [14].

In the present study, all patients underwent standard systematic biopsy followed by radical prostatectomy, providing complete and reliable data for further research. Notably, a high proportion of patients presented with high-risk PCa, as indicated by an average PSA level of 10.3 ng/mL and a mean PI-RADS score of 4.2. Recognizing the potential for reducing biopsy cores in high-risk PCa patients, one of our primary aims was to define the term “regional”. Although various previous studies offered differing interpretations, some defined it as including 6 ipsilateral SB cores based on half-lobe distribution and 4 perilesional cores based on the targeted biopsy lesion [15, 17]. Other studies even proposed sector-based definitions for systematic prostate biopsy [38]. For simplicity and due to widespread familiarity among urologists with systematic core distribution, we opted to define the “regional area” as the 4 systematic biopsy needles closest to the TB needles, facilitating standardization in future research.

We evaluated TrSB’s ability to detect csPCa and ciPCa, as shown in Table 2. Compared to other studies, our research yielded a higher csPCa detection rate (83.5%), likely due to our high-risk patient population who underwent RP. Notably, TrSB missed only 1 csPCa case (0.62%, 1/161), compared to STB’s 11 missed cases (6.8%, 11/161). This suggests that high-risk patients might not require additional systematic needles to diagnose lesion extent through TrSB. Furthermore, TrSB displayed superior accuracy (99.5% vs. 94.3%) and NPV (97% vs. 74.4%) compared to TB alone while showing no significant difference from STB (McNemar test, *p* > 0.999). Conversely, TB exhibited a significant difference from STB (McNemar test, *p* < 0.01), strongly suggesting its inadequacy for PCa patients. While ciPCa results were less encouraging, TrSB still outperformed TB, showing no difference with STB. These findings support the potential of TrSB as a sufficient approach for PCa biopsy. Pathological agreement between biopsy Gleason Group (GG) and RP GG was also promising. Agreement across TB/TrSB/STB was similar (43.3%/46.4%/45.9%), including weighted kappa values (0.48/0.51/0.51). This suggests that different biopsy schemes yield similar agreement between biopsy GG and RP GG. Downgrading and upgrading rates were also comparable across all three schemes. These findings may be explained by the high-risk patient group having their RP GG classified based on the most severe lesion area, potentially overestimating positive treatment post-RP. Consequently, for this group, TB GG might predict RP GG outcomes. Further analysis of the PI-RADS 4/5 subgroup (*n* = 154) in the Supplementary Table yielded similar results: agreement of TB/TrSB/STB remained similar (47.4%/48.1%/46.1%), including weighted kappa values (0.50/0.51/0.50) and grade shift. These positive outcomes suggest that TrSB, including STB, could potentially predict pathological outcomes.

The latest 2023 EAU guidelines on PCa call for more studies on using regional biopsy as the standard approach. Through a retrospective multicenter study of biopsied and surgically treated patients, our team provides evidence that a “regional” approach may be a reasonable way to reduce systematic biopsy for high-risk patients. However, our study has limitations. First, it was a retrospective study. Second, EAU Guidelines indicate transperineal biopsy as first choice, but our study was limited to the transrectal approach, which can lead to complications. Wu Q et al. indicated that transperineal biopsy was more effective than transrectal MRI-TB at detecting PCa and csPCa [40]. Future research should explore the transperineal approach, which may offer a better way to divide the prostate. Additionally, we could not determine whether dividing the prostate into sectors could further reduce SB cores in GG1 patients. Investigating this option holds promise for further minimizing biopsy needs.

## Conclusion

Our new biopsy scheme, TrSB (combining targeted biopsy with the 4 closest systematic biopsy needles), reduces biopsy core count by 8 compared to STB. Notably, TrSB achieves a better detection rate for csPCa than TB alone while maintaining equivalence to STB. Encouragingly, there is no significant difference in pathological agreement between the GG scores obtained through TB, TrSB, and STB when compared to the final diagnosis from radical prostatectomy.

### Electronic supplementary material

Below is the link to the electronic supplementary material.

Supplementary Material 1

## Data Availability

All data were saved well, we are pleased to be contacted when data were needed. Further enquiries can be directed to the corresponding author.
