# Supplementary material for: A novel biopsy scheme for prostate cancer: targeted and regional systematic biopsy
Source: BMC Urol. 2024 Apr 13;24:85. doi: 10.1186/s12894-024-01461-4 (PMC11015685; doi:10.1186/s12894-024-01461-4)
Supplement: Supplementary file 1 — Supplementary Material 1 [file 12894_2024_1461_MOESM1_ESM.docx]

| **Supplementary TABLE 1.** Weighted κ agreement and score changes between RP GG and three biopsy schemes GG. (PI-RADS=4/5, N=154) | | | | | | | | | | | |
| --- | --- | --- | --- | --- | --- | --- | --- | --- | --- | --- | --- |
|  | **RP GG** | | | | |  | **Agreement** | **Weighted κ** | **Downgrade** | **No** | **Upgrade** |
|  | **1** | **2** | **3** | **4** | **5** | **Total** | **(%)** | **(95% CI)** |  | **Change** |  |
| **TB GG** | | |  |  |  |  | 47.4 | 0.50  （0.42-0.58） | 16.2% | 47.4% | 36.4% |
| 0 | 2 | 6 | 1 | 0 | 0 | 9 |  |  | 0 | 0 | 9 |
| 1 | 2 | 10 | 2 | 0 | 0 | 14 |  |  | 0 | 2 | 12 |
| 2 | 0 | 28 | 8 | 3 | 2 | 41 |  |  | 0 | 28 | 13 |
| 3 | 0 | 12 | 24 | 7 | 3 | 46 |  |  | 12 | 24 | 10 |
| 4 | 0 | 3 | 4 | 9 | 12 | 28 |  |  | 7 | 9 | 12 |
| 5 | 0 | 0 | 2 | 4 | 10 | 16 |  |  | 6 | 10 | 0 |
| **TrSB GG** | | |  |  |  |  | 48.1 | 0.51  （0.42-0.59） | 16.9% | 48.1% | 35.0% |
| 0 | 0 | 0 | 0 | 0 | 0 | 0 |  |  | 0 | 0 | 0 |
| 1 | 2 | 13 | 2 | 0 | 0 | 17 |  |  | 0 | 2 | 15 |
| 2 | 2 | 32 | 12 | 4 | 1 | 51 |  |  | 2 | 32 | 17 |
| 3 | 0 | 11 | 21 | 6 | 4 | 42 |  |  | 11 | 21 | 10 |
| 4 | 0 | 3 | 3 | 9 | 12 | 27 |  |  | 6 | 9 | 12 |
| 5 | 0 | 0 | 3 | 4 | 10 | 17 |  |  | 7 | 10 | 0 |
| **STB GG** | | |  |  |  |  | 46.1 | 0.50  （0.42-0.59） | 17.5% | 46.1% | 36.4% |
| 0 | 0 | 0 | 0 | 0 | 0 | 0 |  |  | 0 | 0 | 0 |
| 1 | 2 | 13 | 1 | 0 | 0 | 16 |  |  | 0 | 2 | 14 |
| 2 | 2 | 34 | 14 | 4 | 1 | 55 |  |  | 2 | 34 | 19 |
| 3 | 0 | 10 | 18 | 7 | 4 | 39 |  |  | 10 | 18 | 11 |
| 4 | 0 | 2 | 5 | 7 | 12 | 26 |  |  | 7 | 7 | 12 |
| 5 | 0 | 0 | 3 | 5 | 10 | 18 |  |  | 8 | 10 | 0 |
| RP indicates radical prostatectomy; GG indicates Grade Group; TB indicates targeted biopsy; TrSB indicates targeted and regional systematic biopsy; STB indicates standard biopsy (systematic and targeted); CI indicates confidence interval. | | | | | | | | | | | |
